# From Recurrent Pain to a Life-Threatening Condition: A Case Report of Left Paraduodenal Hernia

**DOI:** 10.7759/cureus.42596

**Published:** 2023-07-28

**Authors:** Ayesha Hina, Muhammad Jawad Zahid, Mahnoor Ataullah Jan, Abdul Haseeb, Musarrat Hussain

**Affiliations:** 1 Department of Surgery, Hayatabad Medical Complex Peshawar, Peshawar, PAK

**Keywords:** surgery, case report, laparotomy, hernia, paraduodenal hernia

## Abstract

Paraduodenal hernia (PDH) is the most frequent type of congenital hernia, especially on the left side. Although acute intestinal obstruction due to internal hernias is rare, it can be life-threatening if not detected and treated promptly. Here, we present the case of a 36-year-old man who presented to the emergency department with an acute intestinal obstruction that had been developing for three days. The patient had a history of recurrent abdominal pain but had never undergone any abdominal surgery. Surgical exploration revealed a left internal PDH with a collection of incarcerated jejunal loops retrocolically encased within the hernial sac. Resection of the gangrenous jejunal loops was performed, followed by hernia reduction and neck closure. This case highlights the importance of timely and accurate diagnosis of left PDHs, particularly in patients with a history of recurrent abdominal pain and no prior abdominal surgeries. Early detection and treatment can prevent life-threatening complications such as intestinal perforation and peritonitis.

## Introduction

Paraduodenal hernias (PDHs) are rare but the most common type of congenital internal peritoneal recess hernia [1]. Its incidence is 53% among the 0.6-5.8% of reported cases of internal hernias [2]. PDH is divided into two subgroups, namely, right PDH and left PDH, with the left being the more common form, also known as Landzert’s hernia [1-3]. The most prevalent type of internal hernia, accounting for 53% of all instances, is the PDH, which accounts for 0.2-0.9% of cases of intestinal obstruction. Regarding gender, internal hernias affect men three times more frequently than women [4].

Radiologists and clinicians are challenged by internal hernias due to their rarity and the complexity of their diagnosis and treatment [5]. The clinical presentation includes chronic non-specific symptoms such as chronic abdominal pain, constipation, and indigestion over time, posing a diagnostic challenge and leading to more acute presentations, such as acute intestinal obstruction, or a more grave presentation, such as intestinal ischemia and perforation and its sequelae if untreated [2,6].

Preoperatively, timely diagnosis is often difficult, leading to grave consequences and a reported mortality rate of more than 20% [2]. Medical history, clinical examination, laboratory diagnostics, and preoperative computed tomography (CT) contribute to the diagnosis. Despite the stated diagnostic possibilities, the final diagnosis is usually made during surgery [6]. The ultimate modality of treatment is operative repair, either laparoscopic or open repair, depending on the operative facilities and level of competency available [3,6]. Laparoscopic surgery has a quicker recovery period, although both procedures have comparable long-term results [7]. In this article, we present a case of a left PDH in a 36-year-old man who arrived at the hospital complaining of severe abdominal pain for the past week that had been exaggerated in the last three days. The main objective of this report is to increase awareness of the likelihood of PDHs as one of the causes of intestinal obstruction. The work has been reported in line with the Surgical CAse REport (SCARE) criteria [8].

## Case presentation

A 36-year-old married man presented to the emergency room with a three-day history of colicky abdominal discomfort, vomiting, and constipation. The pain had started three days prior and gradually worsened over the past week, accompanied by non-projectile bilious vomiting and nausea. He had also experienced a decrease in urine output and was unable to pass stool or flatus. He had no comorbidities, and his drug history consisted of painkillers and laxatives taken for constipation. Moreover, he had no significant family history.

**Figure 1:**
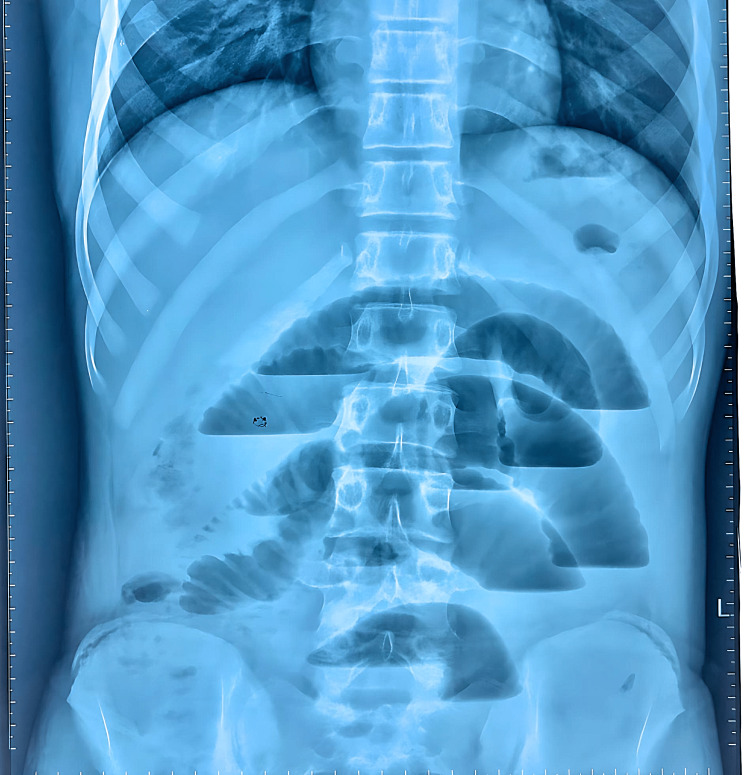
Preoperative erect X-ray of the abdomen showing multiple air-fluid levels indicating small bowel obstruction.

Upon physical examination, the patient had tachycardia, a distended and diffusely tender abdomen, and sluggish bowel sounds. Laboratory investigations revealed an increase in serum amylase with a total leukocyte count of 9.1 × 10^9^/L. Abdominal ultrasound showed gaseous distention of his abdomen and gallbladder sludge. An erect X-ray of the abdomen showed multiple air-fluid levels (Figure 1).

The patient had experienced similar instances of abdominal pain in the past three to four years, which responded to over-the-counter painkillers. He had also been hospitalized once before for severe obstruction. Although his urine output improved with intravenous fluids, his bowel functions did not improve even after 48 hours of resuscitation and treatment.

**Figure 2:**
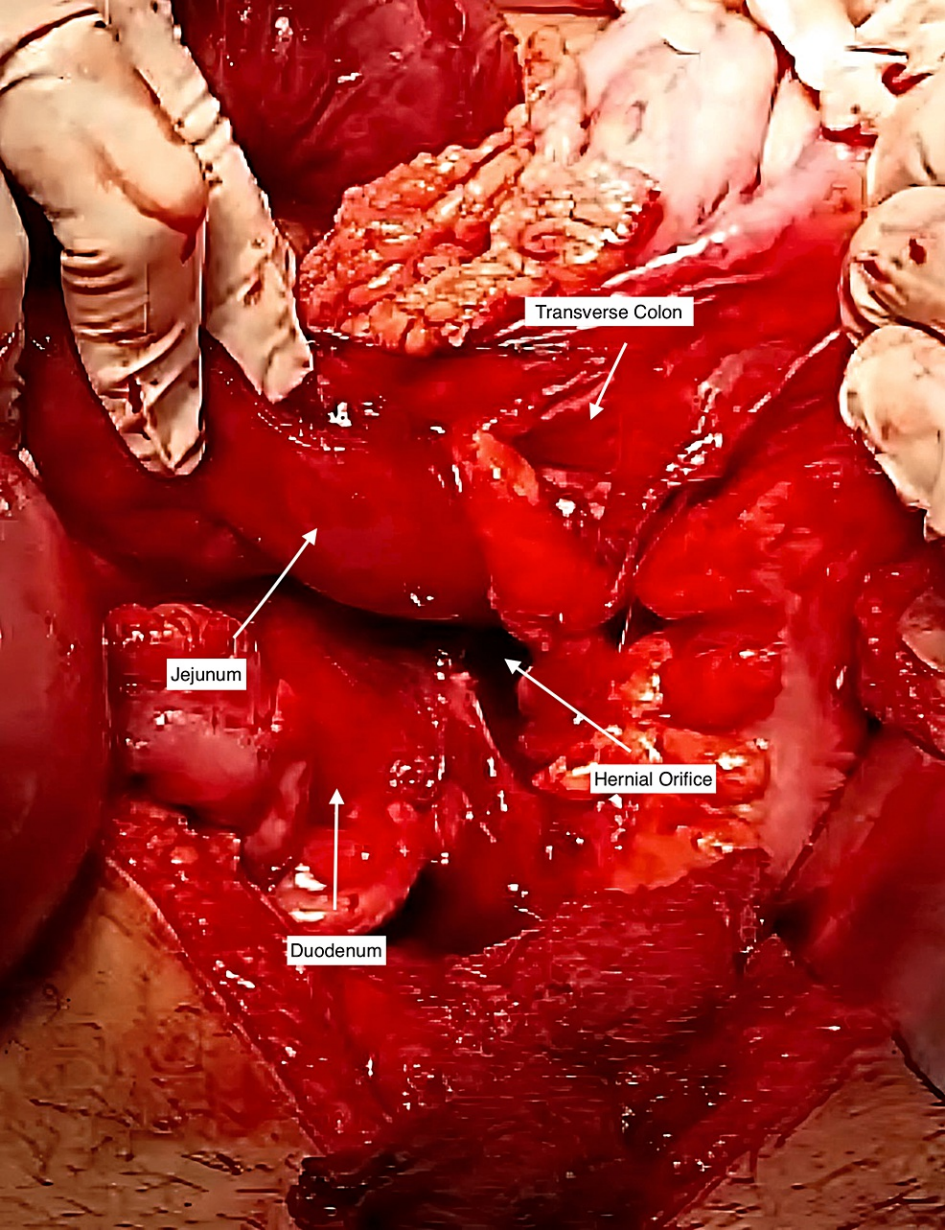
Intraoperative image showing the left paraduodenal space and the neck of the hernia sac.

After three days of evaluation, an exploratory laparotomy was performed by the consultant surgeon due to the patient’s complex and unpredictable condition. The laparotomy revealed hemorrhagic peritoneal fluid and a massively distended gut surrounded by the peritoneal sac and involving the entire abdomen. There was no peritonitis in the abdominal cavity. The left paraduodenal region was found to be covered by a retrocolic collection of small bowel loops encased within the hernial sac to the left of the third part of the duodenum. (Figure 2). An extensive breakdown of adhesions showed that the proximal jejunum had herniated into the mesenteric sac, with almost the entire jejunal loop (almost 6 feet) being gangrenous. The gangrenous portion was resected, and a primary anastomosis was performed, with the mesentery and mesocolon bases approximated. No further abnormalities were found in the peritoneal cavity. The rest of the intestine was examined, which was normal. In addition, all vital organs were normal. The patient’s abdomen was closed in the reverse direction after surgical intervention.

The patient’s postoperative course was uneventful, and he was able to tolerate oral fluids and open his bowels on the third postoperative day. Postoperative laboratory investigations were normal, and he was discharged on the fifth postoperative day in good condition. He was monitored for three months to ensure that the surgical wound had healed and that there were no complications after the procedure.

## Discussion

Internal hernias comprise organ protrusion through a multitude of defects, i.e., normal (foramen of Winslow), paranormal (for instance, the paraduodenal fossa), and abnormal (through a trans-mesenteric, trans-peritoneal, or trans-omental defect). These defects may be congenital due to intestinal malrotation or the absence of retroperitoneal attachments or acquired due to past abdominal surgery, trauma, or any ischemic process [6].

Schizas et al. stated that PDH typically develops between the ages of 40 and 60, while Muneer et al. reported that PDH typically develops at a mean age of 44.1 years [4]. Men experience PDHs three times more frequently than women [5].

The diagnosis of PDHs is the most challenging aspect of management. This is because of the non-specific presentation of PDH. Presenting symptoms can range from diffuse abdominal pain that is frequently eased by positional modifications to acute intestinal obstruction, which is the most common presentation [9]. A contrast CT scan of the abdomen plays a crucial role (accuracy: 95%, sensitivity: 95-100%), and, in most cases, confirms the diagnosis [10]. This is in addition to a thorough medical history, a clinical examination of the patient, an abdominal X-ray, an abdominal ultrasound, laboratory tests, and possibly an angiography of the upper mesenteric artery (displacement of jejunal arteries in the upper left quadrant of the abdomen) [10]. Because our patient did not have adequate resources and financial support to undergo a CT scan, a conservative management approach for spontaneous resolution was initially employed.

The importance of CT in the early preoperative identification of PDH is justified by the significant death rate associated with these complications [11]. The typical CT finding is a mass of dilated small intestinal loops arranged abnormally between the stomach and pancreas to the left of the Trietz ligament. The transverse colon, the duodenal flexure, and the posterior wall of the stomach are frequently displaced due to a mass effect. At the entry of the hernial sac, the mesenteric arteries supplying the herniated small bowel seem congested, engorged, and stretched [12,13].

The ultimate modality of treatment is operative repair, either laparoscopic or open repair, depending on the operative facilities and level of competency available [1,3,6]. There is considerable debate regarding whether to perform a laparoscopy or a laparotomy. The literature does, however, demonstrate that laparoscopic repair of a PDH can reduce hospital stay, encourage early eating, and lower the likelihood of perioperative problems [14,15]. Patients with PDH have a 20-50% mortality rate for acute presentations; hence, early surgical intervention is crucial to prevent subsequent problems. In this case, the delay in surgical intervention caused the gut to become gangrenous and necessitate a resection.

## Conclusions

This case report highlights the importance of early and accurate diagnosis of left PDHs, which can be life-threatening if not detected and treated promptly. The findings of this study demonstrate the necessity of a thorough physical examination and prompt imaging in patients with a history of recurrent abdominal pain and no prior abdominal surgeries. The successful treatment of this patient through laparotomy and resection of the gangrenous portion of the gut, followed by hernia reduction and neck closure, emphasizes the critical role of surgical intervention in such cases. This case report contributes to the existing literature on PDHs and provides valuable insights into the diagnosis and management of this rare condition.
